# Predicting Arts Engagement in Older Adults: A Prospective Analysis Using the MIDUS Study

**DOI:** 10.1093/geroni/igaf122.3072

**Published:** 2025-12-31

**Authors:** Jacquelyn Stephens, Jennifer Smith

**Affiliations:** Mather Institute, Illinois, United States; Mather Institute, Evanston, Illinois, United States

## Abstract

Regular participation in the arts shows promise for increasing older adults’ well-being (Fancourt et al., 2019): Previous work has found associations between arts engagement and increased well-being, as well as reductions in loneliness, depression, and health problems. However, there has been less attention on what factors predict later arts participation in middle- and older age. The current study draws from two waves of the Mid-life in the United States (MIDUS) study, the Refresher 1 (2011-14) and the Refresher Arts Surveys (2021-22), to examine how facets of subjective well-being predict arts engagement over 9 years. The sample (N = 1545), restricted to ages 55+, had an average age of 68.32 (SD = 7.73), was 54.2% female, and 79% white. Predictive factors included Ryff’s six aspects of eudaimonic well-being (WB), life satisfaction (LS), and positive affect (PA). Logistic regression models were used to examine associations between well-being and later monthly arts participation, with covariates of age, gender, education, retirement status, and health. Several aspects of WB predicted greater likelihood of arts participation (purpose in life [OR: 1.03, 95% CI = 1.01, 1.05], personal growth [OR: 1.06, 95% CI = 1.04, 1.08], environmental mastery [OR: 1.02, 95% CI = 1.01, 1.04], and autonomy [OR: 1.03, 95% CI = 1.01, 1.05]), as did LS (OR: 1.19, 95% CI = 1.05, 1.29) and PA (OR: 1.22, 95% CI = 1.02, 1.38). These findings suggest that cultivating well-being may serve as a pathway to arts participation, enhancing quality of life in later years.

